# Decision tree model based prediction of the efficacy of acupuncture in methadone maintenance treatment

**DOI:** 10.3389/fneur.2022.956255

**Published:** 2022-10-06

**Authors:** Yu Dong, Baochao Fan, Enliang Yan, Rouhao Chen, Xiaojing Wei, Jie Zhan, Jingchun Zeng, Hao Wen, Liming Lu

**Affiliations:** ^1^Clinical Research and Big Data Laboratory, South China Research Center for Acupuncture and Moxibustion, Medical College of Acu-Moxi and Rehabilitation, Guangzhou University of Chinese Medicine, Guangzhou, China; ^2^School of Artificial Intelligence, South China Normal University, Guangzhou, China; ^3^Postdoctoral Research Station, Department of Rehabilitation, The Second Affiliated Hospital of Guangzhou University of Chinese Medicine, Guangzhou, China; ^4^Department of Rehabilitation, First Affiliated Hospital of Guangzhou University of Chinese Medicine, Guangzhou, China; ^5^The Third Affiliated Hospital of Guangzhou University of Chinese Medicine, Guangzhou, China

**Keywords:** methadone maintenance treatment, machine learning, decision tree, feature importance, acupuncture

## Abstract

**Background:**

Patients with MMT often face difficulties such as sleep disturbance, headaches, and difficulty in complete abstinence from drugs. Research has shown that acupuncture can mitigate side effects while attenuating methadone dependence. It also has a synergistic and attenuated effect on methadone maintenance treatment (MMT). Exploring the predictors of the efficacy of acupuncture intervention in MMT might help clinicians and patients promote acupuncture-assisted participation in MMT, and improve clinical treatment strategies for MMT.

**Objective:**

To describe the effect of potential predictors on MMT after acupuncture intervention by building a decision-tree model of data from *A Clinical Study of Acupuncture-assisted MMT*.

**Design, setting, and participants:**

In this randomized controlled trial, 135 patients with MMT underwent acupuncture at the Substance Dependence Department of Guangzhou Huiai Hospital in Guangzhou, Guangdong Province, China.

**Intervention:**

A total of 135 patients were 1:1 randomly assigned to either an acupuncture plus routine care group (acupuncture plus methadone) or a routine group (methadone only) for 6 weeks, and followed up for 10 weeks. Sex, age, education level, route of previous opioid use, years of opioid use, and MMT time were recorded before the trial.

**Outcome measurements and statistical analysis:**

All analyses were based on the intention-to-treat (ITT) population. The two decision tree models used the change of methadone dosage and the VAS score for opioid desire as response variables, respectively, and the evaluation criteria were positive effect (decreased by ≥20%) and no effect (decreased by <20%, or increased). We generated the respective feature weights for the decision tree and evaluated the model's accuracy and performance by Precision-Recall.

**Results:**

The overall accuracy of methadone reduction and psychological craving VAS scoring decision trees were 0.63 and 0.74, respectively. The Methadone Dosage Efficacy decision tree identified years of opioid use (weight = 0.348), acupuncture (weight = 0.346), and route of previous opioid use (weight = 0.162) as key features. For the VAS Score decision tree, acupuncture (weight = 0.618), MMT time (weight = 0.235), and age (weight = 0.043) were the important features.

**Conclusion:**

Exploratory decision tree analysis showed that acupuncture, years of opioid use, route of previous opioid use, MMT time, and age were key predictors of the MMT treatment. Thus, acupuncture-assisted MMT strategy should consider the relevant influencing factors mentioned above.

**Patient summary:**

Understanding patient characteristics and the impact of acupuncture regimens on methadone dosage reduction in MMT patients may help physicians determine the best treatment regimen for patients. An analysis of data from our clinical trial showed that acupuncture, years of opioid use, route of previous opioid use, age, and MMT time were key predictors of progressive recovery in patients with MMT. Eligible patients may benefit most from the MMT rehabilitation that reduces consumption and psychological cravings for methadone.

**Clinical trial registration:**

http://www.chictr.org.cn/index.aspx, identifier: ChiCTR1900026357.

## Introduction

Global drug abuse presents several serious challenges. Nearly 275 million people were drug addicts in 2021 (1). In North America, opioid overdoses and deaths have been declared public health emergencies (2, 3). Currently, methadone maintenance treatment (MMT) is the mainstay of treatment for opioid abuse. Patients need to take methadone over the long term, or even for life, which often causes symptoms such as sleep disturbance, dry mouth, sweating, fatigue, constipation, and mental desire (4, 5). Due to the above adverse reactions, methadone patients often take a variety of drugs. Methadone often attenuates the effects of these other drugs, which in turn leads to withdrawal and an increased risk of relapse (6). Thus, patients generally have a strong desire for methadone reduction.

In recent years, extensive research has been conducted on how acupuncture improves the quality of life for patients with MMT, reduces heroin craving (7, 8), and facilitates methadone dose reduction. Additionally, acupuncture-assisted MMT has the advantages of affordability, reliable efficacy, and safety (9). Studies have shown that acupuncture, especially body acupuncture and electroacupuncture, can prolong patients' drug withdrawal time. Moreover, they were two times less likely to relapse than sham groups, and they have also reported mental benefits during withdrawal, such as relief from anxiety, depression, and insomnia (10–12). Acupuncture-assisted MMT can also improve desire, affect expectations, and quality of life for patients taking methadone. It also can facilitate reductions in methadone doses (13, 14).

Our previous studies, and those of other teams, have demonstrated the effects of acupuncture-assisted MMT (3). Yet it remains unclear which acupuncture-based factors influence methadone reduction. Determining this would be of utmost importance in decision-making for acupuncture-assisted MMT treatment. Machine learning offers a way to uncover the relationship between various factors and analyze the associated variables' degree of influence (15). Many machine learning algorithms are black-box theories, and the decision tree model is an important machine learning tool for decision analysis, due to its visualization and interpretability characteristics (16). In this study, we use a decision tree model to analyze data from a randomized controlled trial (RCT) to investigate the effects of acupuncture on MMT. This could help clinicians identify which patients may have varying degrees of influence on treatment outcomes.

For these reasons, this study aims to use a decision tree, a classic algorithm for machine learning, to explore the influence of methadone reduction factors and the relationship between the variables of methadone reduction and craving (acupuncture, age, sex, education level, route of previous opioid use, years of opioid use, and MMT time).

## Materials and methods

### Study design

The data we selected came from our previous clinical RCT, which was a study of acupuncture-assisted methadone reduction. A total of 135 patients with MMT were recruited from the methadone outpatient clinic at the Substance Dependence Department of Guangzhou Huiai Hospital, from October 2019 to September 2020. We randomly divided them into an acupuncture plus routine care group and a routine group (the acupuncture plus routine care group used body acupuncture plus methadone; the routine group used methadone alone). This was done *via* a central randomization system (SAS 9.4) at a 1:1 ratio, and the two groups included 68 and 67 patients, respectively. The trial lasted 6 weeks and was followed up for 10 weeks. Details about the study design for the RCT are provided in the Supplementary materials. We then established decision tree models of daily methadone consumption and visual analog scale (VAS) score, respectively, and generated weights for each feature to evaluate acupuncture's influence on methadone reduction. The protocol was approved by the Ethics Committee at the First Affiliated Hospital of Guangzhou University of Chinese Medicine (No. Y-2019-241), and registered in the Chinese Clinical Trial Registry (ChiCTR1900026357).

### Predictor selection

The dosage of methadone is related to factors such as patients' sex, age, education, and psychological stress (17, 18). Combined with the researchers' judgment on the clinical efficacy and clinical trial design of our previous RCT, we chose acupuncture, age, sex, education level, route of previous opioid use, years of opioid use, and MMT time as our feature index ultimately.

### Outcomes

#### Daily dosage of methadone

Patients' daily methadone consumption from the baseline to the 2nd, 4th, and 6th weeks was recorded on a computer by the prescribing physician at the clinic. We observed changes in methadone dose from baseline to the 6th week.

#### VAS score for methadone craving

Participants were asked to mark their level of craving on a 100 mm line, with 0 representing no craving and 100 representing strong craving (19). VAS scores were recorded from the baseline to the 2nd, 4th, and 6th weeks. We observed changes in VAS scores from baseline to the 6th week.

#### Efficacy evaluation criteria were divided into two categories

Comparison of the differences between methadone dosage and VAS score before and after 6 weeks of treatment; decreased by ≥20% was a positive effect, decreased by <20% or increased was no effect. These classification criteria are based on a guideline that say in the process of methadone reduction, there should be a 5–10 ml reduction within every 1-2 weeks or a reduction of 10% of the current dose. (20). Our RCT lasted 6 weeks. Patients with a 20% reduction in the methadone dosage and the VAS score generally show improvement in sleep status and heart disease relief. Therefore, we chose 20% as the basis for division.

### Statistical analysis

#### Baseline characteristics and results comparison

In this study, all analyses were based on the intention-to-treat (ITT) population. We conducted a descriptive statistical analysis of the description of the baseline characteristics and the comparison of the results. Measurement data were described by means, and to compare the means between the two groups, we used an independent sample *t*-test. Enumeration data were described by frequency, and comparisons of differences between the two groups were assessed with a χ^2^ test. All statistical tests were analyzed in SPSS version 25 (IBM SPSS Statistical Software, New York, USA). *P* < 0.05 (two-sided) was considered statistically significant.

#### Decision tree model

We performed decision tree modeling on data from the ITT population to describe and visualize the impact of potential predictors on methadone dosage based on acupuncture interventions.

In the decision tree, the data set consisted of a series *{(x*_1_*, y*_1_*),(x*_2_*, y*_2_*),…, (x*_*n*_*, y*_*n*_*)}*. Each iteration contains an input *x*_*i*_, which may include multiple characteristics (for example, age, sex, education…) and a class *y*_*i*_, (for example positive or no effects…). This data set is called the training set. Classification tasks must meet the following functions: *f (x*_*i*_*)* = *y*_*i*_, which means each input has a corresponding category. To obtain a high-performance generalization model, *f* needs to satisfy the generalization function. When given a data point *m* that is not in the training set, *f (m)* should output the correct label.

Generating a decision tree is a recursive process that does a good job of breaking up our data, and the basic flow follows a simple “divide and conquer” strategy. The decision tree consists of a root node, several leaf nodes, and several internal nodes. All decision trees start at a root node at the top of the tree, dividing the dataset into a hierarchy of subsets, represented by branch-like segments, and ending with the leaves of the described subsets. The root node contains the complete set of samples, the leaf node corresponds to the decision result, and the path from the root node to each leaf node corresponds to a decision test sequence. The splitting selection of nodes is based on the minimum Gini coefficient to produce the possible attributes and possible values.

A total of 135 people participated in our study. After cleaning and normalizing the data in the database, we took the sex, age, education level, participation in acupuncture treatment or not, years of opioid use, route of previous opioid use, and MMT time as independent variables, and methadone reduction and VAS score (positive or no effect) as dependent variables to establish a database. In the two classification attributes, the data are roughly evenly distributed. In the methadone reduction and VAS score data set, 72 samples and 68 samples had a positive effect, and 63 samples and 67 samples had no effect, respectively. As there was no missing data, the missing value was not filled. We randomly divided the 135 participants into two groups: one to serve as the training set to build the decision tree model (consisting of 80% of the total), and the other to verify the model's accuracy (the other 20%).

**Figure 1:**
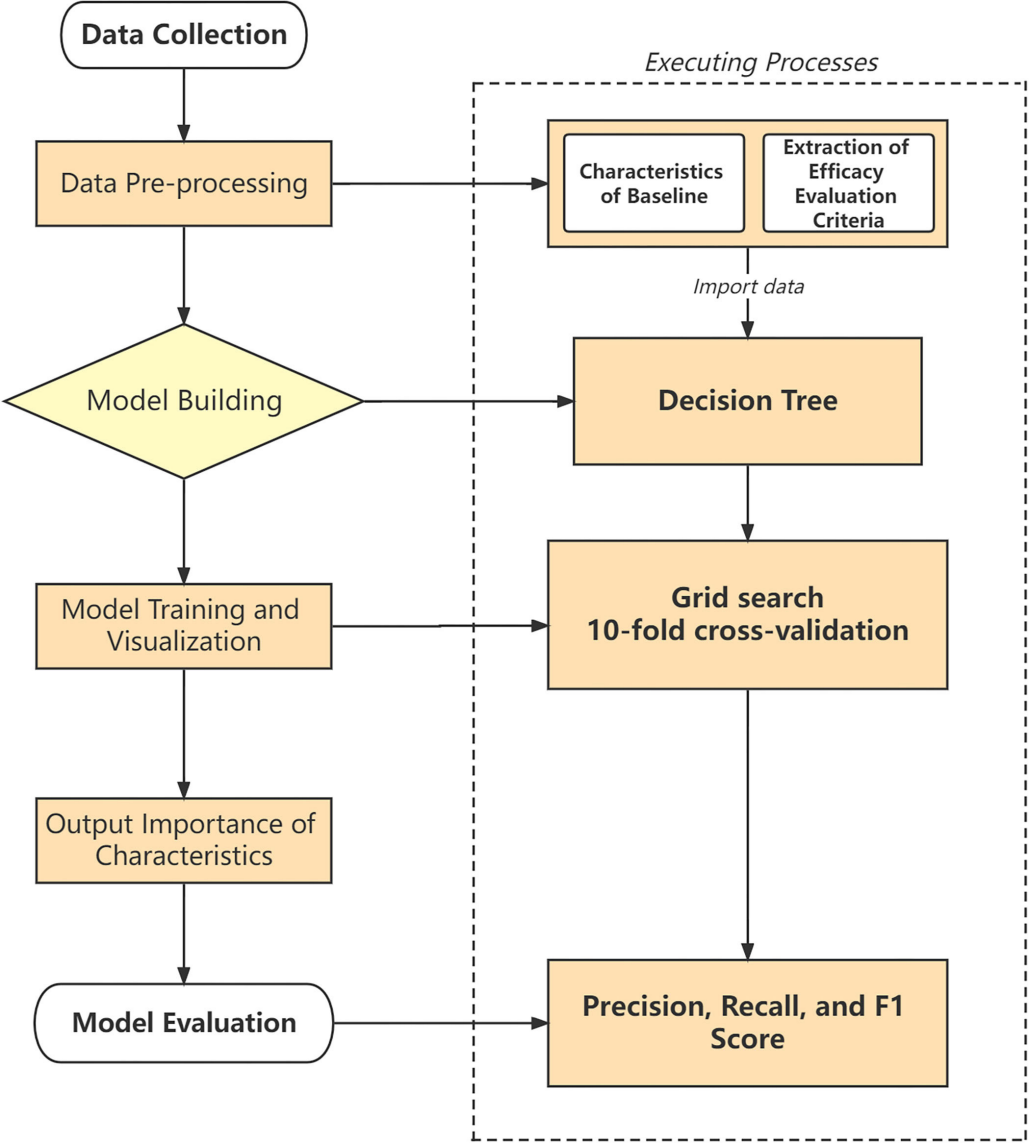
Study design and technical roadmap.

In the process of building a decision tree, we used the grid search method to iterate over the parameters of the decision tree and used the 10-fold cross-validation method to perform multiple verifications, with the highest scoring parameters as the results of the grid search. After the test, we selected Precision, Recall, F-1 score, and Accuracy as indicators to evaluate the decision tree model's performance. Precision is the number of real positive samples among the total positive samples, reflecting the model's ability to predict the accuracy of the positive samples. Recall rate is the samples predicted to be positive and which actually are positive, accounting for all positive samples, reflecting the model's ability to predict the completeness of the positive samples. The *F1* score is the weighted harmonic average of precision and recall. The closer the three metrics are to 1, the better the model performance, and the closer to 0, the worse the model performance. Finally, we obtain the optimal parameter values for the decision tree. Figure 1 shows the study design and technical roadmap. For decision tree construction, we used the scikit-learn Python package version 1.0.1 (21).

## Results

### Patient disposition and baseline characteristics

During the trial period, 135 patients were randomly assigned at a ratio of 1:1 to either an acupuncture plus routine care group or a routine group. The patient baseline characteristics are listed in Table 1. There were no statistically significant differences in age, sex, years of opioid use, MMT time, education, or route of previous opioid use between the two groups (*P* > 0.05). This indicated that the baseline data for the two groups were balanced and comparable.

**Table 1:** Baseline characteristics of the study.

**Variable**	**Acupuncture plus routine care group**	**Routine group**	***P*-value**
	**(*n =* 68)**	**(*n =* 67)**	
**Age**, mean ± SD	51.47 ± 4.72	49.46 ± 8.24	0.401
**Sex (%)**			0.371
Male	55 (80.9)	58 (86.6)	
Female	13 (19.1)	9 (13.4)	
**Years of opioid use (year)**, mean ± SD	16.72 ± 5.86	15.60 ± 8.58	0.246
**MMT time, (year)**, mean ± SD	8.34 ± 3.87	7.13 ± 3.38	0.071
**Education (%)**			0.468
primary school	13 (19.1)	18 (26.9)	
Middle school	28 (41.2)	22 (32.8)	
High school or university	27 (39.7)	27 (40.3)	
**Route of previous opioid use (%)**			0.146
Injection	51 (75)	41 (61.2)	
Nasal	14 (20.6)	18 (26.9)	
Oral	3 (4.4)	8 (11.9)	

SD, standard deviation.

### Outcomes of effectiveness

As shown in Table 2, we used the methadone dosage efficacy and the VAS score efficacy as observational outcomes. They were also the output variables in the decision tree.

**Table 2:** Outcomes of efficacy evaluation criteria.

	**Acupuncture plus routine care group**	**Routine group**	***P*-value**
	**(*****n =*** **68)**	**(*****n =*** **67)**	
**Methadone dosage efficacy (%)**			<0.001
Positive effect	48 (70.6)	24 (35.8)	
No effect	20 (29.4)	43 (64.2)	
**VAS score efficacy (%)**			<0.001
Positive effect	60 (88.2)	8 (11.9)	
No effect	8 (11.8)	59 (88.1)	

#### Methadone dosage efficacy

In the acupuncture plus routine care group, 48 patients (70.6%) had a positive effect of reducing methadone dosage by more than 20% over 6 weeks, while in the routine group, only 24 patients (35.8%) had a positive effect. Twenty (29.4%) patients in the acupuncture plus routine care group did not reduce methadone by more than 20% (no effect). Among the routine group, there were 43 (64.2%) such patients. Statistical analysis of the differences between the two groups showed *P* < 0.001, indicating that the efficacy of methadone reduction was significantly different between the two groups. Additionally, the acupuncture plus routine care group had significantly better efficacy in reducing methadone dosage than the routine group.

#### VAS score efficacy

Sixty patients (88.2%) in the acupuncture plus routine care group had a positive effect (VAS score had decreased more than 20% over 6 weeks), while there were eight of such patients (11.9%) in the routine group. In comparison, eight (11.8%) patients in the acupuncture plus routine care group and 59 (88.1%) patients in the routine group had no effect <a 20% decrease in the VAS score. Statistical analysis showed that there was a statistical difference in the VAS score efficacy between the two groups (*P* < 0.001), and the acupuncture plus routine care group had better efficacy in relieving methadone craving.

### Model performance

#### Model evaluation

We built the decision tree based on the trained model, and the model evaluation results are shown in Table 3.

**Table 3:** P-R value and *F 1* score for each class.

	**Precision**	**Recall**	***F 1* score**	**Accuracy**
**Methadone dosage efficacy**				0.63
Positive effect	0.60	0.69	0.64	
No effect	0.67	0.57	0.62	
**VAS score efficacy**				0.74
Positive effect	0.76	0.81	0.79	
No effect	0.70	0.64	0.67	

We used test sets to evaluate the decision tree model's accuracy. The precision, recall, and *F1* score of the two decision tree models are summarized in Table 3. The test set is the data that has not been utilized in training the decision tree.

The accuracy of the methadone dosage efficacy decision tree model was 0.63, while the VAS score efficacy decision tree model was 0.74.

#### Constructing the decision tree model

**Figure 2:**
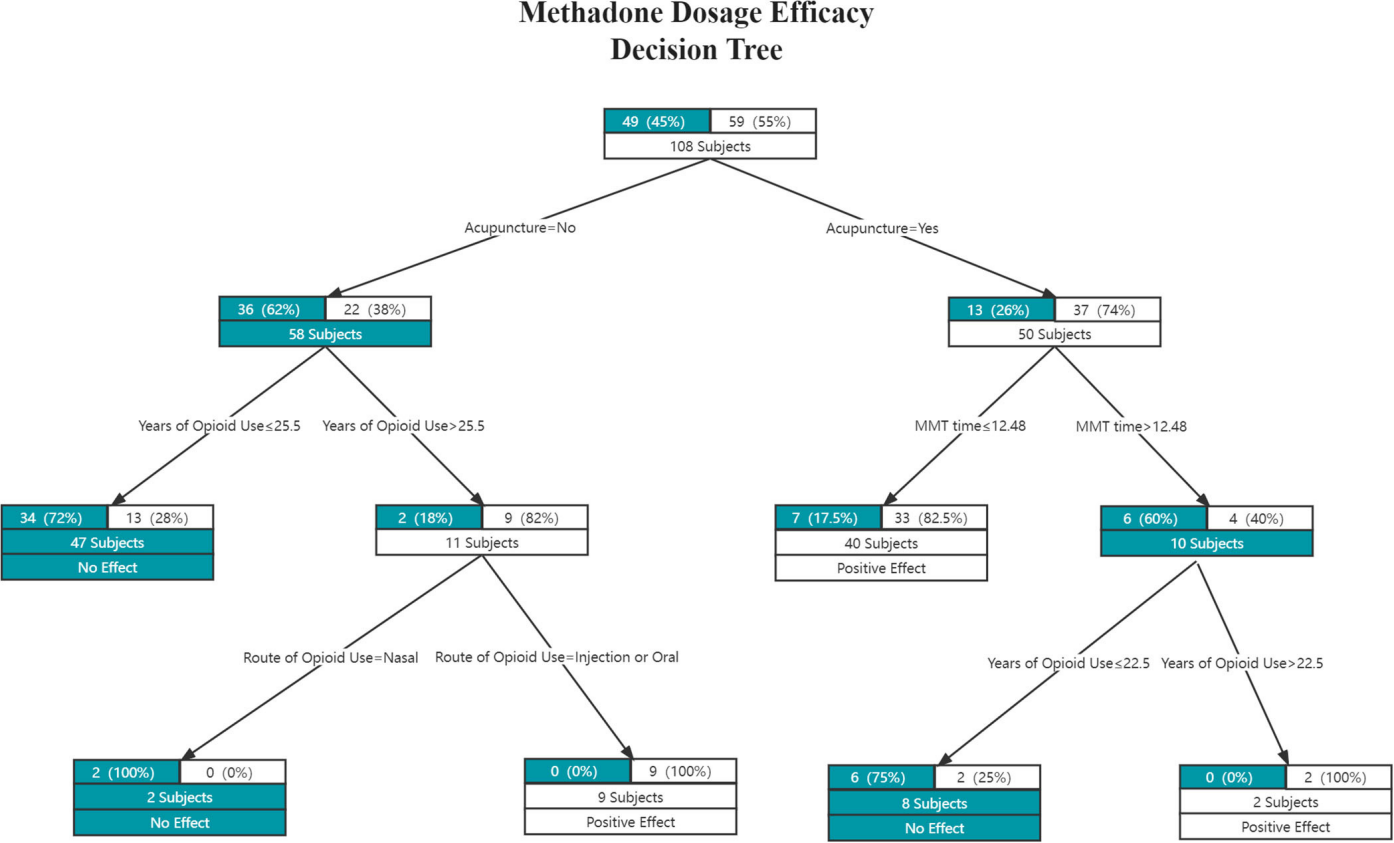
The methadone dosage efficacy decision tree. Each edge represents a classification attribute, and each node contains the number of samples from the previous node up to that point, and their distribution among the two classes. Each leaf node is also labeled with the classification choice (White = positive effect; Gree*n* = no effect).

**Figure 3:**
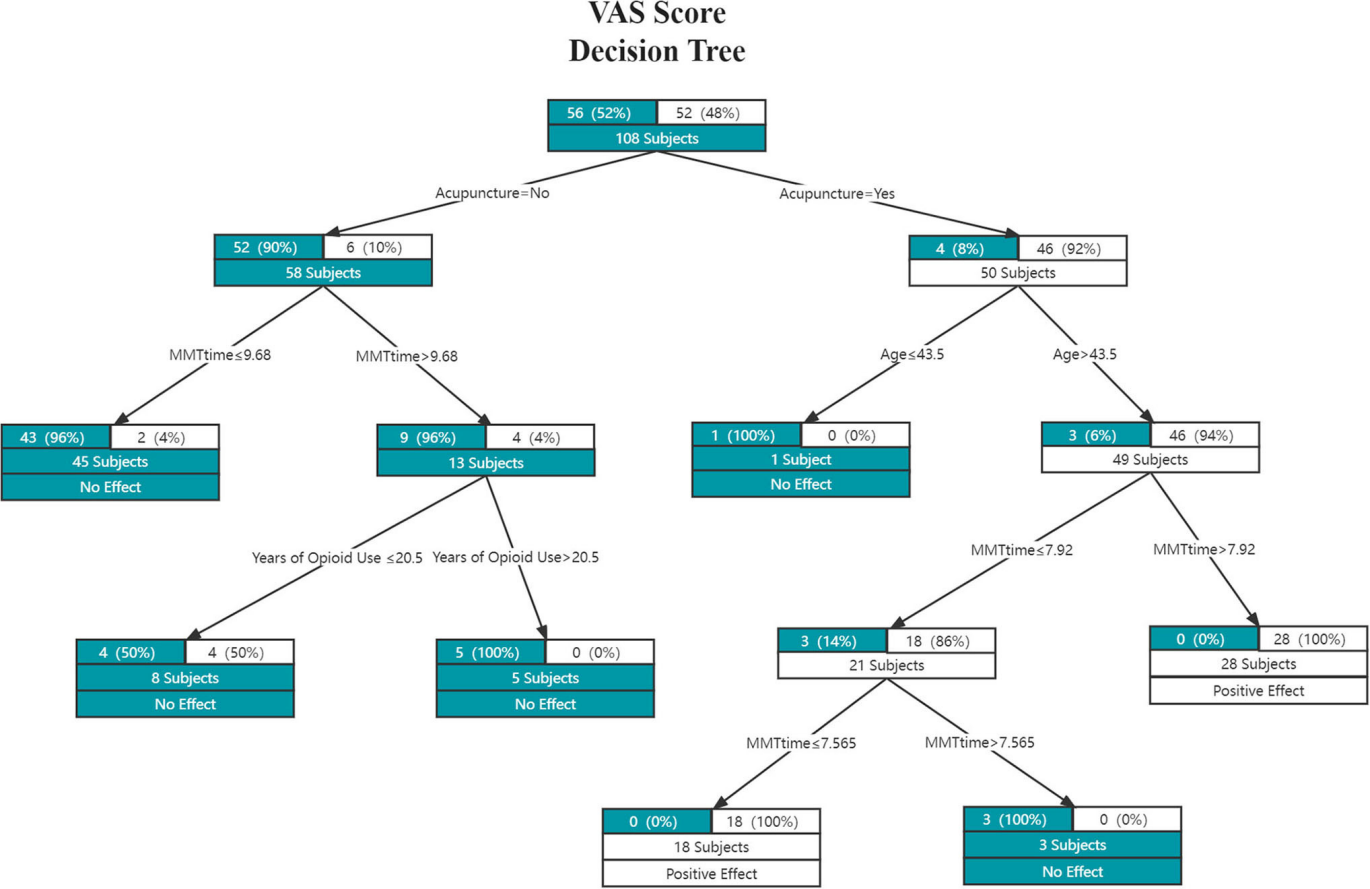
The VAS score efficacy decision tree. Each edge represents a classification attribute, and each node contains the number of samples from the previous node up to that point, and their distribution among the two classes. Each leaf node is also labeled with the classification choice (White = positive effect; Gree*n* = no effect).

As Figures 2, 3 show, the decision trees are constructed in pairs because there are two efficacy criteria based on the effect of acupuncture on the methadone effectiveness index and VAS index. The closer it is to the top of the decision tree, the more important the feature is; the closer it is to the bottom of the decision tree, the less the factors are correlated.

The criteria of the Methadone Dosage Efficacy decision tree model were evaluated by the Gini coefficient, while the VAS score efficacy decision tree was evaluated by the Entropy coefficient. In the methadone reduction model, the maximum depth of the decision tree was 4, and the maximum leaf nodes were 6. In the VAS model, the maximum depth of the decision tree was 5, and the maximum leaf nodes were 7.

As shown in Figure 2, acupuncture can significantly reduce methadone dosage when (1) the MMT time is ≤12.48 years, or (2) the MMT time is > 12.48 years, and opioid use is > 22.5 years. In short, judging from the positive effects of the acupuncture plus routine group, the shorter the MMT time, the easier it is for acupuncture to produce good results; when the MMT time as well as the opioid use time are both longer, the acupuncture can produce a positive effect.

As shown in Figure 3, acupuncture can reduce patients' methadone psychological craving when (1) the patient's age is > 43.5 years and the MMT time is ≤ 7.565 years, or (2) the patient's age is > 43.5 years and the MMT time is > 7.92 years. In general, in the acupuncture plus routine care group, when the patient is older and the MMT time is longer or shorter, acupuncture can produce positive results.

#### Feature importance

**Figure 4:**
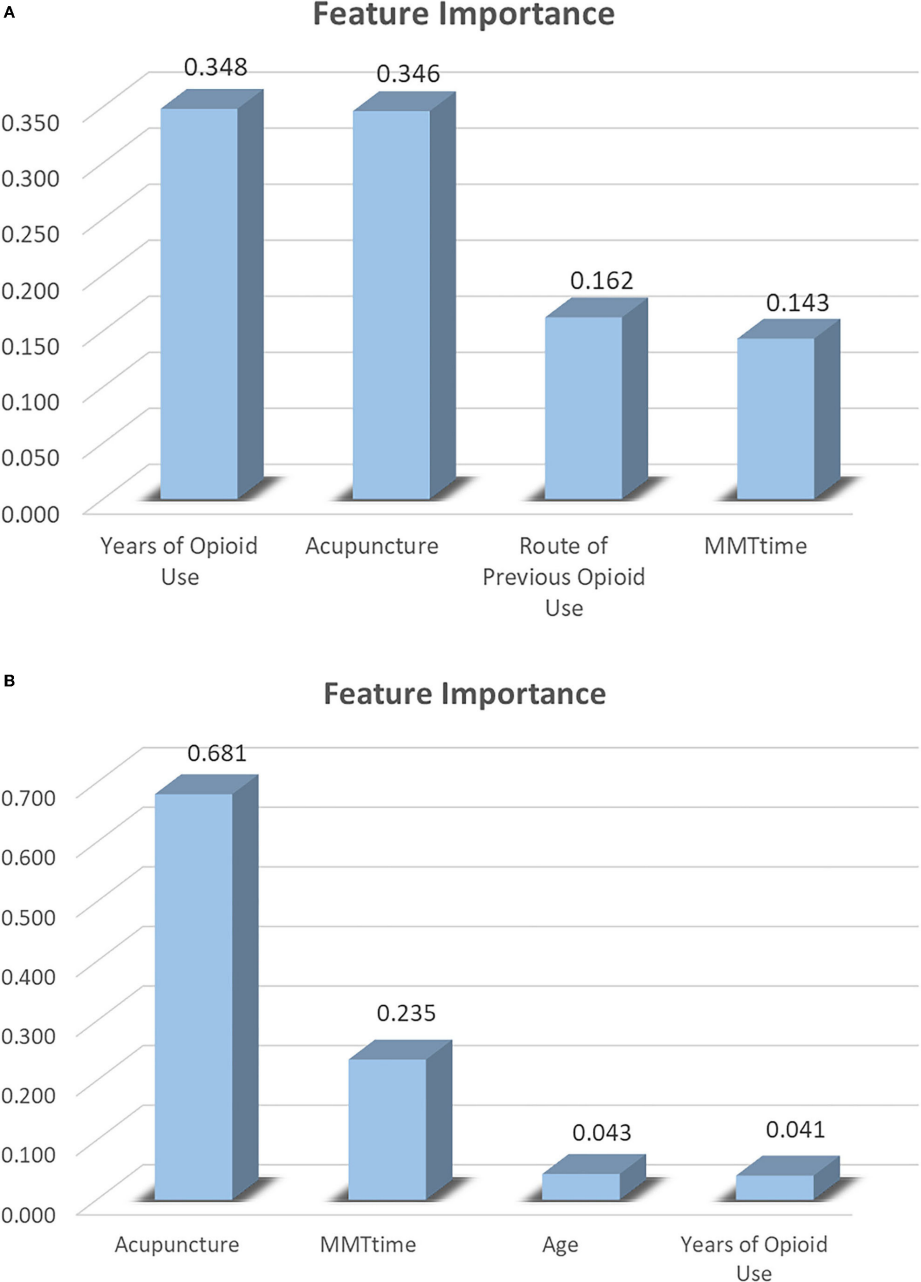
Feature importance. **(A)** Methadone dosage efficacy. **(B)** VAS score efficacy.

Figure 4 shows the factors that influenced the methadone dosage and the VAS score of psychological cravings. Figure 4A summarizes the most significant factors in the methadone dosage efficacy, they are (1) years of opioid use (weight = 0.348); (2) acupuncture (weight = 0.346); (3) route of previous opioid use (weight = 0.162); and (4) MMT time (weight = 0.143). Figure 4B summarizes the feature importance of the attributes that affect the VAS score: (1) acupuncture (weight = 0.681); (2) MMT time (weight = 0.235); (3) age (weight = 0.043); and (4) years of opioid use (weight = 0.041).

## Discussion

This study explores the important role of acupuncture in reducing methadone dosage and psychological cravings in patients with MMT. This is the first time that a decision tree algorithm has been used to explore the importance of acupuncture in the experimental process.

An exploratory decision tree model of this clinical trial data identified acupuncture, opioid use time, route of opioid use, age, and MMT time as common key predictors of recovery from MMT. Patients who meet these criteria appear to benefit the most from acupuncture initiated early in treatment.

In the two decision trees, we selected patients' age, sex, education, opioid use time, route of opioid administration, time on methadone, and whether they participated in acupuncture as predictors. In terms of the importance of feature contribution, the following feature values are all 0: for the characteristics of the methadone reduction index, education, age, and sex; in terms of psychological craving: sex, education, and the previous route of opioid use. They did not appear in Figure 4, suggesting that they did not play an important role in reducing methadone dosage and psychological craving.

In this study, characteristic importance analyzed by the decision tree revealed that the important features of both decision tree models were acupuncture, years of opioid use, and MMT time. We can speculate on the duration of opioid use, the duration of MMT treatment, and whether or not acupuncture has a significant impact on the VAS score and methadone reduction effect. In addition, acupuncture was at the forefront, playing the most important role in reducing patients' psychological craving for methadone, and in reducing methadone dosage. The findings of this study align with those of previous studies. After acupuncture intervention, the daily dose of methadone can be significantly decreased (7), and methadone patients' sleep quality significantly increases (8). At the same time, acupuncture as adjuvant therapy for MMT patients has the advantages of affordability, reliable efficacy, and safety (9). Additionally, body acupuncture plays a greater role than ear acupuncture, electro-acupuncture, transcutaneous electrical stimulation, Western medicine, and traditional Chinese medicine (22). These results are encouraging, as they offer guidance on combining opioid use time and MMT time so that acupuncture can play a greater role. They also provide specific guidance for clinical application.

Our decision tree model offers an upgrade to outcomes produced from a focus on MMT alone. Today, machine learning is widely used to assess opioid overdose risk, and to predict opioid use and relapse (23–25). David et al. established an “extreme random forest” prediction model to provide timely recommendations for local public health interventions to prevent drug overdose deaths (26). Dong et al. achieved high-precision automated predictions to support the healthcare industry in responding to the opioid crisis (27).

In machine learning, there are many ways to meet this requirement. The main reasons we chose the decision tree are: (1) The decision tree algorithm's calculation process is simple and clear, and it can generate easy-to-understand visual images. Unlike Logistic Regression (LR), Naïve Bayes Classification (NBC), Support Vector Machine (SVM), the Stochastic Gradient Boosting method (SGB) (28–31), Artificial Neural Network (ANN), K-Nearest Neighbor (KNN), and other learning algorithms; the decision tree can display the decision-making and classification process for each step. In the other aforementioned algorithms, it is difficult to know the specific training process, leaving researchers with a “black box”. (2) It allows us to assess each feature's importance. In decision tree images, features closer to the root node tend to be more important than those located toward a leaf node. Since the decision tree algorithm always selects the optimal decision in which to split at each node, it plays an important role in predicting the main factors of event occurrence. Adway et al. used a decision tree to explore the influence of the time factor for marijuana use on personal risk (32). Mehdi et al. used decision trees to explore the cost of purchasing drugs, age of first drug use, history of smoking cessation, MMT patients' medication frequency, methadone treatment frequency, and other factors that are associated with drug relapse (33). While most of the above studies have focused on the risks posed by opioids, in our study, we focused on patients' quality of life during MMT and explored the role of acupuncture with the help of a decision tree algorithm.

This study has several advantages over similar studies. First, we conducted a rigorous randomized controlled trial in the study's early stages, in which we obtained real MMT patient data to reflect the status of real individuals undergoing MMT. In addition, our study supplements the outcome measure of acupuncture-assisted methadone treatment reducing psychological craving in patients. We assessed this from both psychological and physical perspectives. Second, this is the first time that machine learning algorithms have been used to analyze methadone use among MMT patients under acupuncture intervention, whereas previous machine learning algorithms have tended to focus on opioid use. We expect that the construction of the decision tree will produce new ideas for rehabilitation management in clinical MMT. Third, this clinical study provides a new treatment for opioid overdose, suggesting a focus on clinical treatment. At the same time, the decision tree technique analyzed and verified the therapeutic value of acupuncture in the treatment of MMT patients, and the character analysis also suggested other important roles affecting MMT efficacy.

However, this study has several limitations. First, due to the small sample size and the lack of specific quantitative relationships between acupuncture and MMT time and opioid use time, the decision tree model in this study is exploratory. Thus, it can only facilitate the exploration of the approximate distribution of each predictor. Second, in this study, there was no comparison of the outcomes of multiple acupuncture methods (such as electro-acupuncture, ear acupuncture, etc.), acupuncture time, or acupuncture frequency differences. In future research, the acupuncture process could be further refined to rectify this shortcoming. Third, MMT patients are often plagued by sleep disturbance, anxiety, depression, etc., and these ailments also affect treatment efficacy in MMT patients. This was not included in our study. In the future, machine learning algorithms could also be used to explore acupuncture treatment of these diseases in greater depth. In future research, we plan to conduct a larger sample, treatment, and longer follow-up trial to confirm the clinical efficacy of acupuncture-assisted MMT.

## Conclusion

This trial and decision tree algorithm demonstrated that acupuncture therapy showed a positive effect on methadone reduction and psychological craving in MMT patients. Additionally, the decision tree model based on the VAS score efficacy was highly accurate. This provides reference suggestions for MMT clinics to promote the application of acupuncture- and MMT-related guidelines.

## Data availability statement

The raw data supporting the conclusions of this article will be made available by the authors, without undue reservation.

## Ethics statement

The studies involving human participants were reviewed and approved by the Ethics Committee of the First affiliated Hospital of Guangzhou University of Chinese Medicine. The patients/participants provided their written informed consent to participate in this study.

## Author contributions

EY, JZe, HW, and LL contributed to the conception and design of the study. YD drafted the manuscript. EY, LL, HW, and BF contributed to the critical revision of the article for important intellectual content. HW, XW, JZe, RC, and JZh performed the acupuncture treatment for patients. YD and BF analyzed the data. XW and RC collected and registered data. All authors contributed to manuscript revision, read, and approved the submitted version.

## Funding

This study was funded by the National Natural Science Foundation of China (82174527), the Guangdong Provincial Key Fields of Higher Education (the New Generation Information Technology) in 2020 (No. 2020ZDZX3024), the Guangzhou University of Traditional Chinese Medicine Double First-Class and the High-level University Discipline Collaborative Innovation Team (2021xk55), the China Postdoctoral Science Foundation (2021M703779), and China Postdoctoral Science Foundation (No. 2021M690797).

## Conflict of interest

The authors declare that the research was conducted in the absence of any commercial or financial relationships that could be construed as a potential conflict of interest. The reviewer FQ declared a shared affiliation with the author HW to the handling editor at the time of review.

## Publisher's note

All claims expressed in this article are solely those of the authors and do not necessarily represent those of their affiliated organizations, or those of the publisher, the editors and the reviewers. Any product that may be evaluated in this article, or claim that may be made by its manufacturer, is not guaranteed or endorsed by the publisher.
