# Supplementary material for: Decision tree model based prediction of the efficacy of acupuncture in methadone maintenance treatment
Source: Front Neurol. 2022 Oct 6;13:956255. doi: 10.3389/fneur.2022.956255 (PMC9582273; doi:10.3389/fneur.2022.956255)
Supplement: Supplementary file 1 [file Data_Sheet_1.pdf]

Supplementary Material

Decision Tree Model-based Prediction of The Efficacy of Acupuncture in Methadone Maintenance Treatment

Yu Dong(董昱)[1 †], Baochao Fan(范宝超)[1 †], Enliang Yan(闫恩亮)[1, 2], Rouhao Chen(陈柔皓)[1], Xiaojing Wei(魏晓菁)[1], Jie Zhan(詹杰)[3], Jingchun Zeng(曾婧纯)[4*], Hao Wen(文豪)[5*], Liming Lu(陆丽明)[1*]

Contents

1 Details about the Randomized Controlled Trial
2 Acupuncture Point Selection

1 Details about the Randomized Controlled Trial

All patients with methadone maintenance treatment (MMT) included in this study were from Guangzhou, Guangdong Province, China, and were treated at the methadone outpatient clinic of the Substance Dependence Department of Guangzhou Huiai Hospital. Investigators recruited patients at the clinic from October 2019 to September 2020. Interested patients were screened by doctors, and patients who meet the inclusion criteria obtain written informed consent after consulting a doctor. Included patients were divided into an acupuncture plus routine care group and a routine group by a central randomized system (SAS 9.4) in a 1:1 ratio. The trial has been approved by the Ethics Committee of the First Affiliated Hospital of Guangzhou University of Chinese Medicine (No.: Y-2019-241) and registered in the China Clinical Trials Registration (ChiCTR1900026357).

Subjects are required to fulfill the criteria for the diagnosis of opioid dependence based on the Diagnostic and Statistical Manual of Mental Disorders, 5th edition (DSM-V) [1] .In addition, they should also be satisfied the following requirements:(1) male or female aged 18–60 years; (2) receive MMT for more than 30 days; (3) do not receive any kind of acupuncture therapy during the previous 3 months; and (4) can sign informed consent. Participants (1) with serious heart, liver, lung, or kidney disease; (2) venereal disease or AIDS; (3) the presence of severe digestive disease and athrepsia; (4) major psychosis; (5) the receipt of other treatment that may affect the efficacy evaluation of the present intervention; (6) an infection, inflammation, scar or injury close to the site of the selected acupoints; (7) or are pregnant or planning to become pregnant were excluded from the study. Each Participant was recorded before the trial for age, sex, route of previous opioid use, years of opioid use, and MMT time.

Patients were randomly assigned to an acupuncture group or routine group. Acupuncture plus routine care group was treated with acupuncture plus MMT. Acupuncture is performed by an acupuncturist with 3 years of clinical experience. The

selected acupoint groups were derived from Jin's three-needle acupuncture (JTN), which usually takes three to four groups of acupoints and has been used to treat a variety of diseases in recent years. In this study, we chose three acupoint groups, named *Dingshen-zhen, Sishen-zhen,* and *Shouzhi-zhen*. The acupuncture was performed three times a week, retained for 30 min, and twirled every 10 min. Sterile stainless-steel disposable acupuncture needles (Huatuo, Suzhou, China; lengths and diameters 0.3 mm× 25mm or 0.3mm× 40 mm) were used in this group. All patients in the routine group received MMT only. The trial lasted 6 weeks and was followed up for 10 weeks.

The primary outcome measure for drug craving was VAS. Based on previous studies [2] [3], after 4 weeks of treatment, the VAS for drug craving score difference between the baseline and the endpoint in the sham acupuncture group should be approximately 20 ± 30, while the true acupuncture group should be approximately 37.5 ± 28.Type 1 error was assumed at 0.05, and type 2 error was assumed at 0.1. PASS11.0 software (CSS Statistical Software, Kaysville, UT, USA) was used to determine the sample size, and the minimum sample size was 60 subjects for each group. Considering a dropout rate of 15%, a total of 140 subjects was required with 1:1 allocation to each group (70 participants per group) for this study.

2 Acupuncture Point Selection

Acupuncture Point Selection

Acupuncture points	Description
GV21: Qianding *(Sishen-I)*	On the head, 3.5 B-cun superior to the anterior hairline,on the anterior median line.
GV19: Houding *(Sishen-II)*	On the head, 5.5 B-cun superior to the posterior hairline, on the posterior median line.
GV20: Baihui	On the head, 5B-cun superior to the anterior hairline, on the anterior median line.
Sishen-III	On the head, 1.5cun left lateral to the anterior median line and at the same level as GV20.
Sishen-IV	On the head, 1.5cun right lateral to the anterior median line and at the same level as GV20.
EX-HN3:Yintang	On the head, between the right medial end of the eyebrow and the left one.
Dingshen-I	On the head, directly 0.5cun superior to EX-HN3.
GB14: Yangbai	On the head, 1B-cun superior to the eyebrow, directly superior to the centre of the pupil.
Dingshen-II	On the head, directly 0.5cun superior to left GB14.
Dingshen-III	On the head, directly 0.5cun superior to right GB14.
HT7: Shenmen	On the anteromedial aspect of the wrist, radial to the flexor carpi ulnaris tendon, on the palmar wrist crease.

PC6: Neiguan	On the anterior aspect of the forearm, between the tendons of the palmaris longus and the flexor carpi radialis, 2B-cun proximal to the palmar wrist crease.
PC8: Laogong	On the palm of the hand, in the depression between the second and third metacarpal bones, proximal to the metacarpophalangeal joints.

Note: The specified acupoints are from the WHO Standard Acupuncture Point Locations in the Western Pacific Region

REFERENCES

[1] American Psychiatric Association. Diagnostic and Statistical Manual of Mental Disorders (DSM-V). Arlington: American Psychiatric Association; 2013.

[2] Chan YY, Lo WY, Li TC, Shen LJ, Yang SN, Chen YH, Lin JG. Clinical efficacy of acupuncture as an adjunct to methadone treatment services for heroin addicts: a randomized controlled trial. Am J Chin Med. 2014;42(3):569–86.

[3] Fudala PJ, Bridge TP, Herbert S, Williford WO, Chiang CN, Jones K, Collins J, Raisch D, Casadonte P, Goldsmith RJ, et al. Office-based treatment of opiate addiction with a sublingual-tablet formulation of buprenorphine and naloxone. N Engl J Med. 2003;349(10):949–58.
